# Comparison of three commercial multi‐energy CT and electron density phantoms for iodine quantification and tissue substitute characterization

**DOI:** 10.1002/acm2.70735

**Published:** 2026-08-06

**Authors:** Artur Omar, Robert Vorbau, Jens Zimmerman, Gavin Poludniowski

**Affiliations:** ^1^ Medical Radiation Physics and Nuclear Medicine Karolinska University Hospital Stockholm Sweden; ^2^ Department of Clinical Science Intervention and Technology Karolinska Institutet Stockholm Sweden; ^3^ Department of Oncology‐Pathology Karolinska Institutet Stockholm Sweden

**Keywords:** CT number calibration, iodine quantification, multi‐energy CT, stoichiometric calibrations

## Abstract

**Background:**

Multi‐energy computed tomography (MECT) and electron density (ED) phantoms are designed for evaluating the performance of CT scanners. They include substitute materials representing the energy‐ and material‐dependent CT numbers of different organs, tissues, or contrast agents. While MECT phantoms are intended for dual‐energy CT (DECT) and photon‐counting CT (PCCT) applications, ED phantoms are meant for the calibration of CT numbers to a metric relevant for radiation therapy treatment planning.

**Purpose:**

To compare three standard commercial phantoms in terms of iodine quantification for diagnostic imaging and tissue substitute characterization for radiation therapy. The phantoms investigated include the Multi‐Energy CT/Advanced Electron Density Phantom (AED) by Sun Nuclear Corporation (Melbourne, FL, USA), the CIRS Model 662/062M phantom (Norfolk, VA, USA), and the newly introduced Multi‐Energy QA Phantom by QRM (Möhrendorf, Germany), for which we report the first evaluation results.

**Methods:**

The phantoms were imaged using a GE Revolution Apex DECT scanner (GE Healthcare, Waukesha, WI, USA), a Siemens SOMATOM X.ceed DECT scanner (Siemens Healthineers, Forchheim, Germany), and a Siemens NAEOTOM Alpha PCCT scanner. Iodine‐in‐water inserts were evaluated using virtual monochromatic images and iodine maps, whereas the tissue equivalence of four insert sets (AED, CIRS‐ED, CIRS‐EPTN, and QRM) was theoretically assessed. For this purpose, vendor‐specified elemental compositions and physical mass densities were used to derive relative electron density (RED), effective atomic number (EAN), mean excitation energy (I‐value), and mass density (MD), which were then compared with corresponding human tissues listed in ICRU Report 44. CT scans of the phantoms containing these tissue substitutes were subsequently used for CT number calibration to proton stopping power‐ratio (SPR), RED, and MD.

**Results:**

Fitted slopes for reconstructed iodine concentrations were accurate to within 8% for the QRM and AED phantom inserts. For the best performing scanner (PCCT), the slope was within 3%. The CIRS iodine‐in‐water inserts showed substantially higher deviations of up to 22%. Linear attenuation coefficients derived from 70 keV virtual monochromatic images generally agreed with theoretically expected values to within about 10% for the QRM and AED iodine inserts (for concentrations of 1 mg/mL or higher). Regarding the QRM, AED and CIRS‐EPTN tissue substitute sets: they were closely tissue‐equivalent in terms of RED, EAN, I‐value (and hence SPR), but not in terms of MD. The CIRS‐ED inserts were not, however, closely tissue‐equivalent in terms of EAN. Consequently, the QRM, AED and CIRS‐EPTN substitutes satisfy the consensus guide criteria of the European proton therapy community for CT calibration, whereas the CIRS‐ED set does not.

**Conclusion:**

The commercially available phantoms of Sun Nuclear (AED) and QRM are suitable for both MECT performance assessment and CT‐calibration for radiation therapy. However, the batch of CIRS iodine‐in‐water inserts used in this study was unsuitable for MECT assessment. Also, the tissue substitute set provided with the CIRS 062M ED phantom was less tissue‐equivalent than the other sets evaluated.

## 1 INTRODUCTION

Multi‐energy computed tomography (MECT) and electron density (ED) phantoms are designed to account for the energy‐ and material‐dependent x‐ray attenuation of different organs and endogenous materials, such as soft tissue, fat, blood, and calcium, as well as contrast agents like iodine and gadolinium (in dedicated applications). This makes these phantoms ideal for assessing the capabilities of spectral CT scanners, including dual‐energy CT (DECT) and photon‐counting CT (PCCT). The accuracy required for the reference phantom materials also renders them useful for the calibration of CT numbers expressed in Hounsfield units (HU) for radiation therapy treatment planning systems (TPS).

MECT/ED phantoms are usually made of water‐equivalent plastic (solid water) and include several openings to accommodate different phantom inserts. The selection of inserts depends on the intended application. Given that iodine is the predominant contrast medium used clinically, inserts with different concentrations of iodine are considered essential for assessing MECT performance.^1^ AAPM Task Group Report 299 (TG299)^2^ recommends the use of iodine inserts for regular quality control of CT number accuracy in virtual monochromatic images (VMI), material quantitative accuracy (iodine maps), the accuracy of material removed/suppressed images (“virtual noncontrast”; VNC), and accuracy in differentiation tasks (iodine vs calcium). The report further suggests regular testing of CT number accuracy using tissue‐equivalent inserts if the scanner is intended for treatment planning.

In radiation therapy, phantoms with precisely specified tissue‐equivalent inserts are used to evaluate the correspondence between CT number and a dosimetrically relevant quantity. This quantity may be relative electron density (RED), stopping power ratio (SPR) or mass density (MD), depending on the type of therapy, the TPS and the selected dose calculation algorithm. To establish this relationship and define a look‐up‐table (LUT), the inserts should preferably have mass density and chemical composition that match those of human tissues. Furthermore, they should cover the full range of relevant materials, from inflated lung tissue to adipose and soft tissues, extending to high‐density cortical bone.^3^

Several commercial MECT/ED phantoms satisfy the aforementioned criteria for phantom materials.^2^, ^3^ There are also various kinds of user‐customized (homemade) phantoms that have been utilized in research.^4^, ^5^, ^6^, ^7^ Nonetheless, standardized commercial phantoms are generally preferred for routine use because they are manufactured more consistently, remain stable over time, and, importantly, enable comparisons of results across different institutions.^2^ However, it is possible that some discrepancies may arise when comparing results obtained using different commercial phantoms, as the dimensions and compositions of the inserts and the water‐equivalent plastic phantom body are often unique and proprietary to each manufacturer. In addition, insert composition may vary on a phantom‐to‐phantom basis even within the same manufacturer.

This study aims to compare three standard commercial MECT/ED phantoms in terms of iodine quantification for diagnostic imaging and tissue substitute characterization for radiation therapy. The phantoms investigated include the popular Multi‐Energy CT/Advanced Electron Density Phantom by Sun Nuclear Corporation (Melbourne, FL, USA), the CIRS Model 662/062M phantom (originally developed by CIRS now part of Sun Nuclear), and the newly introduced Multi‐Energy QA Phantom developed by QRM (Möhrendorf, Germany), for which we report the first evaluation results. These phantoms are tested in various scenarios using a PCCT scanner and two conventional CT scanners with dual‐energy functionality.

## 2 METHODS

### 2.1 Phantoms

**FIGURE 1:**
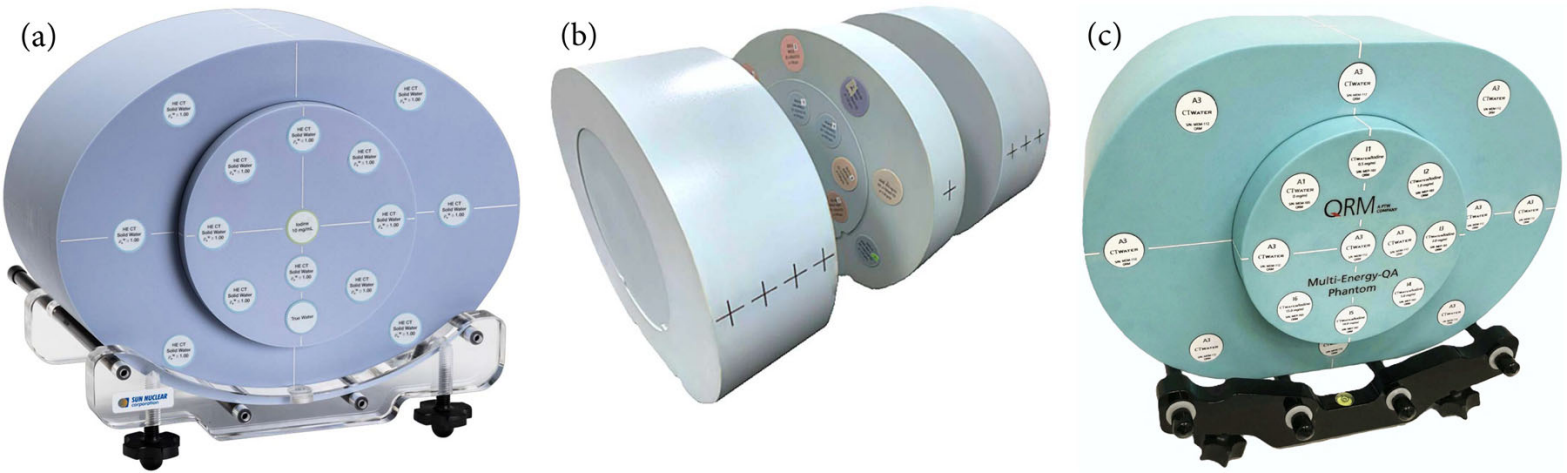
Phantoms investigated in this study: (a) Sun Nuclear Multi‐Energy CT/Advanced Electron Density Phantom, (b) CIRS Model 662/062M, and (c) QRM Multi‐Energy QA Phantom. Insert arrangements are described in the Methods section.

This section overviews the investigated phantoms (shown in Figure 1), including their configurations, insert materials, and other relevant features.

#### 2.1.1 AED

The Sun Nuclear Multi‐Energy CT Phantom and Advanced Electron Density Phantom share the same design and differ only in the accompanying insert sets; hereafter, this phantom family is referred to as the AED phantom for simplicity. It has a water‐equivalent plastic base with a mass density of approximately 1.02 g/${\mathrm{cm}}^{3}$ and a relative electron density of 1.00±0.005. The base measures 40 cm by 30 cm and includes a detachable 20 cm diameter inner cylinder. The phantom is 16.5 cm long, and the inserts span the full length. It accommodates 16 removable inserts (10 in the inner section and 6 in the outer section), each with a diameter of 2.8 cm. The inserts investigated in this study, relevant for TPS calibration and MECT quality control, are listed in Table 1.

**TABLE 1:** Tissue‐equivalent and iodine‐in‐water inserts used with the Sun Nuclear Multi‐Energy CT/Advanced Electron Density Phantom (AED), the QRM Multi‐Energy QA Phantom, and the CIRS Model 662/062M phantom (standard tissue substitutes, CIRS; and European Particle Therapy Network‐recommended substitutes, CIRS EPTN).^3^

	AED	CIRS	CIRS EPTN	QRM
Lung	Lung LN300	Lung Inhale	Lung Inhale	Lung LD
tissues	Lung LN450	Lung Exhale	Lung Medium	ICRU Lung (infl.)
				Lung HD
Adipose	HE Gen Adipose	Adipose	30% Gland 70% Adipose	ICRU Adipose
tissues	HE Breast 50/50	Breast 50/50	70% Gland 30% Adipose	ICRU Breast 50/50
			Gland	
Soft	Liquid Water	Liquid Water	Liquid Water	Liquid Water
tissues	HE CT Solid Water^a^	Plastic Water‐LR^a^	Plastic Water‐LR^a^	CTwater^a^
	HE Brain	Muscle	Soft Tissue Grey	ICRU Brain
	HE Liver	Liver	Spinal Cord	ICRU Muscle
			Kidney	ICRU Liver
			Prostate	ICRU Cartilage
			Brain Tissue	
Bone	HE Inner Bone	200 mg/ml HA	Spinal Disk	ICRU Spongiosa
tissues	CaCO3 30%	800 mg/ml HA	Trabecular Bone	ICRU Femur
	CaCO3 50%	1250 mg/ml HA	30% Cort. 70% Trabec.	ICRU Humerus
	HE Cortical Bone		70% Cort. 30% Trabec.	ICRU Mandible
			Cortical Bone	ICRU Cortical
Iodine	0.2, 0.5, 1, 2, 5,	0.2, 0.5, 1, 2, 5,	—	0.5, 1, 2, 5
(mg/mL)	10, 15, 20	10, 15		10, 15

^a^ Vendor designations for solid water variants.

#### 2.1.2 CIRS

The CIRS Multi‐Energy CT QA Phantom (Model 662) and the CIRS Electron Density Phantom (Model 062M) share the same design, but differ in the specific inserts they contain. The former is equipped with inserts intended for MECT quality control, whereas the latter comes with tissue‐equivalent inserts intended for TPS calibration. These phantoms are made of water‐equivalent plastic with a mass density of 1.029 g/${\mathrm{cm}}^{3}$ and a relative electron density of 0.998. They share the same geometry: the base measures 33 cm by 27 cm and includes a detachable 18 cm diameter inner cylinder. Each phantom has a 5 cm long central region designed to hold the inserts. It can hold a total of 17 inserts (9 in the inner section and 8 in the outer section), each with a diameter of 3 cm. The Model 662 phantom is supplied with extension plates to extend the length to 25 cm. The Model 062M is not supplied as standard with such plates, but some form of extension is recommended for radiotherapy CT calibration.^3^

The inserts used in this study consist of two sets of tissue‐equivalent substitutes, as listed in Table 1. One set (CIRS‐EPTN) corresponds to the substitutes recommended by the European Particle Therapy Network (EPTN),^3^ while the other (CIRS‐ED) comprises the standard set provided with the CIRS Model 062M phantom. Additionally, iodine‐in‐water inserts from the CIRS Model 662 phantom were used, along with iodine‐in‐blood inserts (0.5, 2, 5, 10, and 15 mg/mL) to substantiate the iodine‐in‐water results. Each iodine insert has a small core, 1 cm in diameter, containing the specified iodine concentrations.

#### 2.1.3 QRM

The newly introduced Multi‐Energy QA Phantom from QRM is made of water‐equivalent plastic with a mass density of approximately 1.03 g/${\mathrm{cm}}^{3}$ and a relative electron density of 1.00±0.002. This phantom has dimensions of 25 cm by 35 cm, and extends 12.5 cm in length. It includes a detachable 16 cm diameter inner cylindrical section. The phantom is designed to accommodate a total of 19 inserts (10 in the inner section and 9 in the outer section), each with a diameter of 2.5 cm and covering the full length of the phantom.

The inserts used in this study consist of the base set provided with the phantom, which includes solid‐water inserts with iodine concentrations of 0.5, 1, 2, 5, 10, and 15 mg/mL. Additionally, the optional set of tissue‐equivalent substitutes, designed according to ICRU Report 44 specifications,^8^ was included (see Table 1).

### 2.2 CT imaging

CT images of the phantoms described in the previous section were acquired using a GE Revolution Apex DECT scanner (GE Healthcare, Waukesha, WI, USA), a Siemens SOMATOM X.ceed DECT scanner (Siemens Healthineers, Forchheim, Germany), and a Siemens NAEOTOM Alpha PCCT scanner. These scanners employ different MECT technologies, which can affect the quality of the synthesized images.^9^ The GE Apex scanner uses fast kV switching.^10^ The Siemens X.ceed scanner uses TwinBeam (split‐filter in the longitudinal direction).^11^ The Siemens Alpha scanner uses photon‐counting detectors with multiple energy thresholds.^12^

The phantoms were imaged using two setups: one involving the inner cylindrical section of the phantoms, approximately representing an adult head in size (hence often referred to as the head section), and another involving the full size of the phantoms, approximately representing an adult abdomen in size (hence often referred to as the body section). The same scanning and reconstruction parameters were applied for both configurations, as detailed in Table 2. The parameters generally align with the consensus guide for CT number calibration in proton therapy,^3^ and are appropriate for tissue‐substitute characterization; they were also considered clinically relevant for iodine quantification, except for the dose setting.^2^ A higher dose than that used in routine contrast‐enhanced diagnostic CT was selected to suppress image noise and improve measurement precision, particularly for the low‐iodine concentration inserts. This choice reflects that this study evaluates phantom material properties rather than scanner performance under typical clinical dose conditions.

**TABLE 2:** Scan and reconstruction parameters for the image acquisitions performed in this study, employing a GE Revolution Apex scanner, a Siemens SOMATOM X.ceed scanner, and a Siemens NAEOTOM Alpha scanner.

	Apex	X.ceed	Alpha
**General parameters**			
software version	23MW24.44	VB20A	VB10A
tube current (mA)	365	286	380
rotation time (s)	1	1	0.5
collimation (mm)	40.0	38.4	57.6
helical pitch	0.516	0.500	0.500
DFOV (mm)	350	350	350
slice width (mm)	2.5	2	2
recon. kernel	Standard	Qr40	Qr40
recon. iterative	ASiR‐V 60%	ADMIRE3	QIR3
artifact reduction	—	iBHC	iBHC
CTDI_vol,32_ (mGy)	30	30	30
**Single‐energy scan parameters**			
tube voltage (kV)	120	120	—
tube filter	Large	W1	—
**Multi‐energy scan parameters**			
tube voltage (kV)	140/80	140	120
tube filter	Large	W1+AuSn	W1

For the tissue substitute characterization, the image reconstructions consisted of conventional polychromatic images for the DECT‐capable scanners and 70 keV virtual monochromatic images for the PCCT scanner. Single‐energy CT acquisitions were used despite DECT‐capability being available as the use of polychromatic images is still the clinical norm. For the PCCT scanner, however, the use of virtual monochromatic images was logical as they are available by default. Energy selections between 70 and 80 keV have been used elsewhere for CT calibration.^3^, ^13^ For iodine quantification, virtual monochromatic images were considered, along with iodine maps that measure contrast in mg/mL.

### 2.3 Iodine quantification

The iodine inserts were arranged in the inner section of each phantom, starting with the lowest concentration at the 12 o'clock position and progressing to higher concentrations in a clockwise manner. This arrangement was used both for the setup that included only the inner cylindrical section of the phantoms (the head) and for the setup involving the full size of the phantoms (the body).

The CT numbers of the various iodine inserts were determined from the acquired images by defining a volume of interest covering half of each insert's diameter and its full length, excluding a few initial and final image slices. The analyzed metrics were the mean value and its standard uncertainty (error of the mean). In addition, the combined standard uncertainty was calculated by incorporating both the error of the mean and the uncertainty of the measurement setup. The latter being assessed through an extra series of CT scans in which the setup was reset between each scan.

Quantitative accuracy was assessed in terms of the difference between measured and specified iodine concentrations and by linear regression of measured (iodine‐map) versus specified insert concentrations, with the slope (k) and coefficient of determination ($R^{2}$) reported. Measured CT numbers in virtual monochromatic images were also compared with theoretically expected values for monochromatic energy E and iodine concentration $\rho_{x}$ (in units of g/${\mathrm{cm}}^{3}$), as given by:

(1) $$\mathrm{HU}\left(E;\rho_{x}\right)=1000\;\frac{\mu_{I}-\mu_{w}}{\mu_{w}-\mu_{a}}\left(\frac{\rho_{x}}{\rho_{I}}\right),$$

where $\rho_{I}$ is the elemental density of iodine, while $\mu_{a}$, $\mu_{I}$, and $\mu_{w}$ are the photon energy‐dependent linear attenuation coefficients of air, iodine, and water, respectively. The numerical values applied to these parameters were sourced from the materials database of the PENELOPE Monte Carlo system.^14^ These same values were also used to convert measured CT numbers into linear attenuation coefficients, allowing for the calculation of the percent error compared with the calculated expected values.

### 2.4 Tissue substitute characterization

Theoretical evaluations of the four tissue substitute sets (AED, CIRS‐ED, CIRS‐EPTN, QRM) were performed by taking the stated mass density (MD) and calculating the relative electron density (RED), effective atomic number (EAN) and mean excitation energy (I‐value) for each substitute, given the vendor‐provided elemental compositions. These were compared to theoretical adult human tissues using 32 tissues tabulated in ICRU reports 44 and 46,^8^, ^15^ ranging in density from inflated lung to cortical bone. For estimation of EAN, Mayneord's power law was employed with a power of m=3.1.^16^ Material I‐value was calculated using Bragg additivity and modified elemental I‐values, following the methodology of ICRU report 37.^17^ Tissue equivalence for each substitute set was assessed for each parameter (e.g. EAN or I‐value) by the mean absolute error (MAE) between the values for the ICRU tissues and the values for the substitute sets, interpolating where necessary.

CT calibration for radiation therapy was evaluated by generating look‐up‐tables (LUTs) for proton relative stopping power (at 100 MeV kinetic energy), as well as for RED and MD. Methodologies for LUT generation may be direct (between measured CT numbers of inserts and an estimated quantity), ^18^ stoichiometric (based on fitting for a material set and predicting CT numbers for human tissues) ^19^ or rely on a combination of both (hybrid approach).^3^, ^20^, ^21^ In this work, the Python version of the open‐source consensus guide software ^3^ was used for LUT generation. The 2025 update of the software uses a hybrid calibration for LUT generation of SPR and RED and a pure stoichiometric approach for MD. The stoichiometric model implemented is equivalent to the non‐linear two‐parameter model of Schneider *et al.* 2000.^22^ We extended the Python code to enable production of direct or pure stoichiometric LUTs for all three quantities, as well as the possibility of using an alternative stoichiometric model that has been shown to be more robust to the presence of high atomic number materials in substitutes.^23^

Input data were obtained by scanning the four sets of tissue substitutes (AED, CIRS‐ED, CIRS‐EPTN, QRM) in the corresponding phantoms, in both head and body configurations. Following the consensus guide, multiple soft‐tissue inserts were scanned simultaneously, whereas each bone substitute was scanned individually (positioned in the central cavity of the phantom). We note that the pure “cortical bone” insert was not included in the available CIRS‐EPTN set, with the “70% Cort. 30% Trabec.” insert being the densest available. Regions of interest (ROI) were manually centered in the relevant inserts in the resulting images and the mean values extracted using a Python script. The ROI diameter was set as 16 mm, which corresponded to greater than 50% and less than 70% of the insert diameter, depending on the substitute set and particular insert. A central z‐axis extent of 1 cm was used for data extraction, corresponding to either 4 or 5 slices. For analysis, the “average” output LUTs of the consensus software were used, corresponding to LUTs generated using the average of the head and body CT numbers for each insert.

## 3 RESULTS

### 3.1 Iodine quantification

**FIGURE 2:**
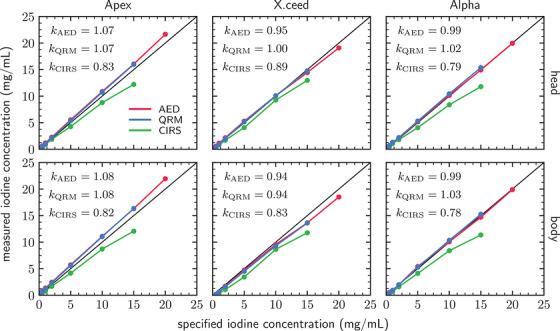
Iodine concentrations specified for iodine‐in‐water phantom inserts compared with the values measured from synthetic iodine maps produced by the GE Revolution Apex (DECT) and Siemens SOMATOM X.ceed (DECT) scanners, and the Siemens NAEOTOM Alpha (PCCT) scanner. The measurements pertain to the head and body configurations of the phantoms investigated. The diagonal indicates unity agreement. For each case, the slope (k) of the linear regression across the available insert concentrations is reported but not the (close to zero) intercept value.

**FIGURE 3:**
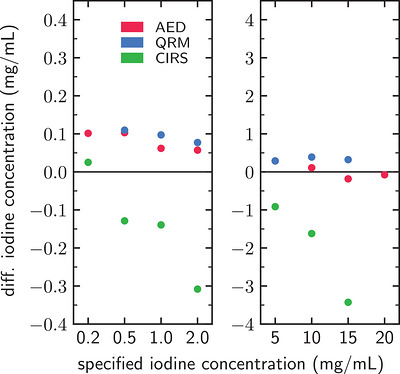
Difference between measured and specified iodine concentrations for different iodine‐in‐water phantom inserts, average over the two phantom sizes. The measurements were obtained from synthetic iodine maps produced by the Siemens NAEOTOM Alpha scanner.

Figures 2 and 3 show that the AED and QRM iodine‐in‐water inserts exhibit a strong linear response with increasing iodine concentration, with $R^{2}>0.99$ across all scanners and phantom configurations. For the GE Apex DECT scanner, the measured concentrations are slightly higher than specified (reflecting a positive bias; fitted slopes k=1.07–1.08), whereas for the Siemens X.ceed DECT scanner, they are slightly lower (reflecting a negative bias; k=0.94–1.00). The PCCT scanner yields slopes closest to unity for the AED and QRM inserts (k=0.99–1.03). For this scanner, the mean absolute error (MAE) is low in both configurations: in the body configuration it is 0.1 mg/mL for AED and 0.2 mg/mL for QRM; in the head configuration, it is 0.1 mg/mL for AED and 0.3 mg/mL for QRM.

By comparison, the CIRS inserts exhibit pronounced deviations, with MAE of 1.0 mg/mL (body) and 0.9 mg/mL (head). The error increases with iodine concentration, reaching a maximum absolute error of 3.6 mg/mL. Although the CIRS response remains linear ($R^{2}>0.99$), the fitted slopes (k=0.78–0.89) indicate systematic underestimation. These concentration errors should be interpreted relative to the combined standard uncertainty, estimated to be less than 0.08 mg/mL across all concentrations, scanners, and phantom configurations investigated.

**FIGURE 4:**
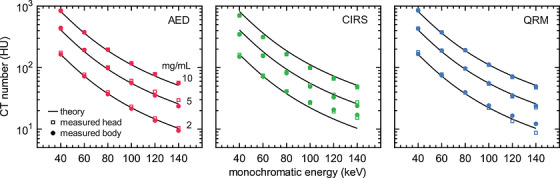
Mean CT numbers of iodine‐in‐water inserts (2–10 mg/mL) measured in 40–140 keV virtual monochromatic images from the Siemens NAEOTOM Alpha scanner, shown for the head and body phantom configurations and compared with theoretical reference values.

In order to assess the energy dependence of the iodine‐in‐water inserts, CT numbers measured in virtual monochromatic images were compared with theoretical reference values calculated using Equation (1), as shown in Figure 4. Overall, the AED and QRM inserts closely follow the theoretical trend. In the body configuration, the MAE is 5.9 HU for AED and 5.3 HU for QRM; in the head configuration, it is 7.7 HU for AED and 6.7 HU for QRM, with a combined standard uncertainty of at most 2 HU across all measurement points. Expressed in terms of linear attenuation coefficients, the mean absolute percentage error (MAPE) is 4.4% for AED and 4.5% for QRM in the body configuration, and 6.1% for AED and 5.7% for QRM in the head configuration. Notably, the agreement improves by about one percentage point at lower energies (40–60 keV), which are most relevant for contrast‐enhanced imaging.

By contrast, the CIRS inserts show considerably larger discrepancies, with MAE of 19.7 HU in the body configuration and 18.9 HU in the head configuration. In terms of derived linear attenuation coefficients, the corresponding MAPE is 14.3% in the body configuration and 12.4% in the head configuration.

Table 3 provides a more detailed comparison of derived linear attenuation coefficients and measured CT numbers in 70 keV virtual monochromatic images with the corresponding theoretical reference values. Across scanners, the maximum absolute deviation in attenuation is about 8% for the AED inserts and 12% for the QRM inserts. In contrast, the CIRS inserts show systematically lower attenuation than expected, with deviations of up to about 23%, corresponding to an error of ‐84 HU. Restricting the analysis to the PCCT scanner (Alpha), the AED and QRM attenuation coefficients agree more closely with theory, remaining within 7% overall and within about 3.5% for the 10–15 mg/mL inserts.

**TABLE 3:** Percent error of the linear attenuation coefficients for iodine‐in‐water inserts relative to theoretical reference values, reported as relative percent error Δμ/μ (%).

	Iodine	AED		CIRS		QRM	
Scanner	mg/mL	Δμ/μ	ΔHU	Δμ/μ	ΔHU	Δμ/μ	ΔHU
Apex	1	−3.4	−0.9	5.6	1.5	12	3.3
	2	2.0	1.0	−1.3	−0.7	9.1	4.8
	5	6.7	8.6	−14	−16	6.8	8.8
	10	5.4	14	−11	−29	5.5	14
	15	3.7	15	−18	−71	6.0	24
X.ceed	1	2.0	0.6	−3.8	−1.0	6.8	1.8
	2	5.6	3.0	−9.2	−5.1	4.9	2.6
	5	7.5	9.8	−18	−23	2.3	3.0
	10	3.8	9.7	−15	−37	0.4	1.1
	15	−0.7	−2.7	−22	−84	0.0	−0.2
Alpha	1	−0.5	0.3	10	4.2	6.8	2.6
	2	2.2	1.2	3.9	2.1	4.5	2.3
	5	5.9	7.7	−13	−17	3.1	3.9
	10	3.5	9.1	−14	−34	2.4	6.1
	15	1.1	4.3	−21	−78	2.9	12

*Note*: The corresponding absolute error in CT number is reported as ΔHU (HU). The results are averaged across head and body phantom configurations and represent 70‐keV virtual monochromatic images from the GE Revolution Apex (DECT), Siemens SOMATOM X.ceed (DECT), and Siemens NAEOTOM Alpha (PCCT) scanners.

**FIGURE 5:**
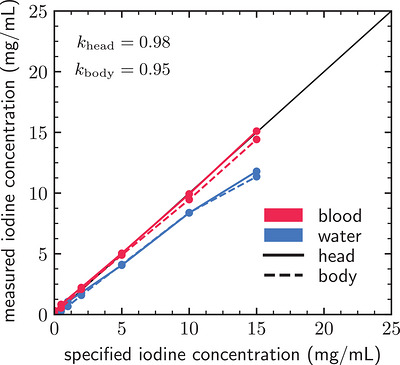
Iodine concentrations specified for CIRS iodine‐in‐blood and iodine‐in‐water phantom inserts compared with the values measured from synthetic iodine maps produced by the Siemens NAEOTOM Alpha scanner. The measurements pertain to the head and body configurations of the CIRS Model 662/062M phantom. The diagonal indicates unity agreement. For each case, the slope (k) of the linear regression across the available iodine‐in‐blood concentrations is reported but not the (close to zero) intercept value.

The results presented so far indicate that the CIRS inserts contain markedly less iodine than intended, with the discrepancy being most pronounced for inserts with higher specified concentrations. To assess whether this issue is limited to the iodine‐in‐water inserts, a second set of iodine‐in‐blood inserts supplied with the CIRS phantom was analyzed. Figure 5 therefore compares the iodine concentrations specified for the different sets of CIRS inserts with the corresponding concentrations measured in the synthetic iodine maps produced by the PCCT scanner.

The figure demonstrates that the iodine‐in‐blood inserts closely match the expected values, with fitted slopes nearly equal to unity (ranging from 0.95 to 0.98). This agreement is observed for both phantom configurations, confirming the accuracy of the CIRS iodine‐in‐blood inserts and sharply contrasting with the performance of the iodine‐in‐water inserts.

### 3.2 Tissue substitute characterization

A direct CT calibration requires close tissue equivalency in: (i) relative electron density (RED) and effective atomic number (EAN)—for RED calibration; (ii) RED, EAN, and I‐value—for SPR calibration; or (iii) RED, EAN, and mass density (MD)—for MD calibration. The stoichiometric approach, however, is designed to correct for non‐tissue equivalency. Yet, in practice, even for such stoichiometric calibrations, approximate equivalency is an advantage. Note that the consensus guide implements a hybrid of direct and stoichiometric methods for SPR and RED calibrations and therefore close tissue‐equivalence of the tissue substitute set is also an explicit requirement.

**FIGURE 6:**
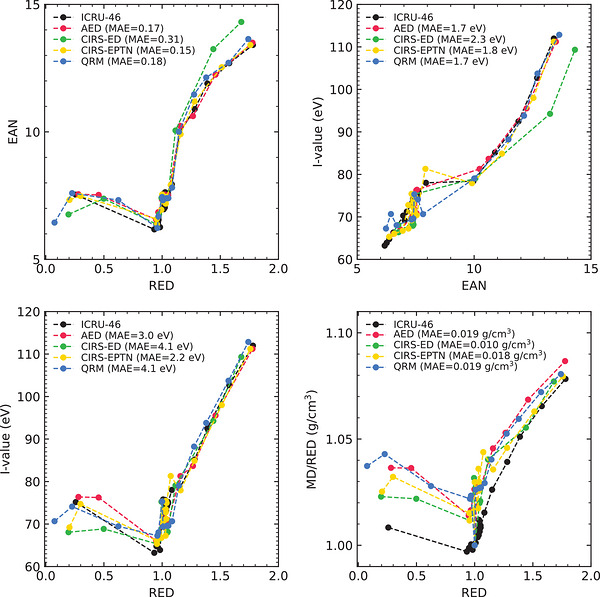
Tissue equivalence of four tissue substitute sets compared to 32 tissues in ICRU report 46.^15^ Effective atomic number (EAN) is plotted against relative electron density (RED) (top‐left); mean excitation energy (I‐value) is plotted against EAN (top‐right); I‐value is plotted against RED (bottom‐left); scaled mass density (MD/RED) is plotted against RED (bottom‐right). The mean absolute errors (MAE) to ICRU tissues are indicated in the legends. MAE‐values were derived using 1‐dimensional linear interpolation of the substitute values to the *x*‐variate values (RED or EAN) of the ICRU tissues.

Figure 6 presents the theoretical tissue equivalence of the four sets of tissue substitutes, based on vendor‐specified compositions and physical densities, with the 32 human tissues from ICRU report 46 as reference. The four sets of substitutes all follow the broad trends of the ICRU adult human tissues. However, compared to the other sets, the CIRS‐ED substitutes exhibit an EAN less consistent with the real tissues, for a given RED or I‐value. For example, for EAN against RED, the CIRS‐ED set exhibits a mean absolute error of 0.31 atomic number units, compared to 0.17 for the AED set. However, all the sets of substitutes exhibit a bias in scaled mass density, with values higher on average by 0.01 to 0.02 g/${\mathrm{cm}}^{3}$ compared to ICRU tissues.

**FIGURE 7:**
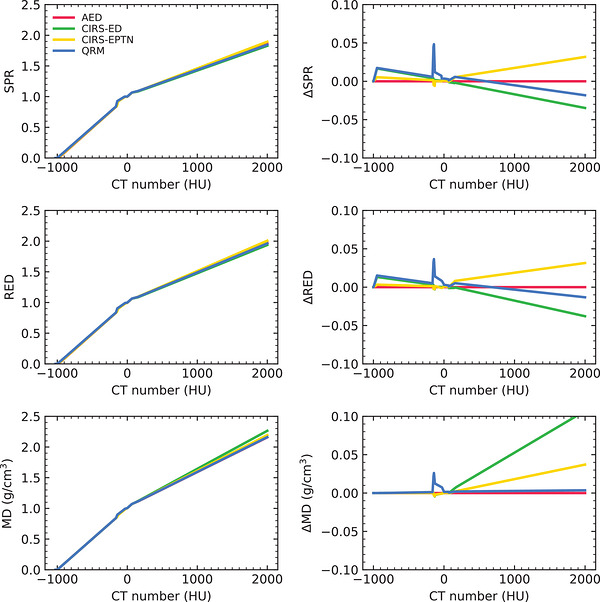
CT calibration look‐up‐tables (LUTs) for proton stopping power ratio (SPR), relative electron density (RED) and mass density (MD) generated using the consensus guide software. Results are for 70 keV virtual monoenergetic images from the Siemens NAEOTOM Alpha scanner and the four tissue substitute sets. The calibrations are for the average CT numbers of each substitute over the two phantom sizes. The left column display the LUTs and the right column the absolute difference to a reference (AED phantom results).

Figure 7 (left column) shows LUTs produced with the consensus guide software for SPR, RED and MD. Results are presented only for the NAEOTOM Alpha scanner, based on 70 keV images. The trends were the same for the Apex and X.ceed scanners and those results are provided in the supplementary materials (see the Figures S1 and S2). Figure 7 (right column) also shows differences compared to the LUTs generated with the AED substitute set. We emphasize that the differences are expressed as absolute discrepancies in the relevant quantity rather than as percentages. The ΔSPR and ΔRED are within 0.05 of the reference in all cases, even up to a CT number of 2000 HU. However, for ΔMD and the CIRS‐ED substitutes, the discrepancy exceeds 0.1 g/${\mathrm{cm}}^{3}$. Note that the spikes in the difference curves below ‐100 HU are due to how the consensus software generates LUTs. The LUT knot points are not set at pre‐determined CT numbers but rather depend on the phantom data. The spike in LUT differences then arises from the abrupt way in which the lung and adipose sections of the LUT are spliced together, with the transitions located at slightly differing CT numbers depending on the phantom.

**FIGURE 8:**
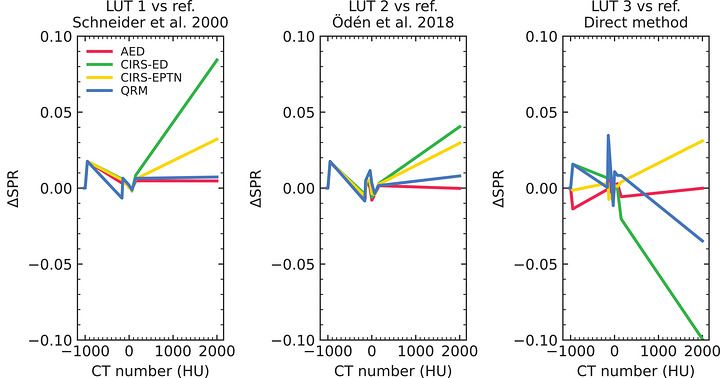
Absolute differences in three CT‐to‐SPR look‐up‐tables compared to a reference of the AED phantom and hybrid LUT. Results are shown for two purely stoichiometric calibrations with different stoichiometric models. The stoichiometric models correspond to Schneider et al.^22^ that is implemented in the consensus guide (LUT 1; left panel) and an alternative of Ödén et al.^23^ (LUT 2; middle panel); results with direct (non‐stoichiometric) calibrations are also provided (LUT 3; right panel). Results are for 70 keV virtual monoenergetic images from the Siemens NAEOTOM Alpha scanner and the four tissue substitute sets.

To illustrate the consequences of different models for LUT generation, Figure 8 shows ΔSPR for three alternative methods: two stoichiometric models and a direct calibration. The consensus hybrid approach and the AED substitutes serve as the reference. Only the CIRS‐ED substitute set, which is the least tissue equivalent, exhibits a high sensitivity to calibration approach; the discrepancy with respect to reference at the upper end of the range (2000 HU) varied from ΔSPR = 0.08 (LUT 1) to ΔSPR = −0.1 (LUT 3).

## 4 DISCUSSION

### 4.1 Iodine quantification

Iodine‐in‐water inserts spanning multiple concentrations have been evaluated to assess their suitability for iodine‐related performance studies and routine MECT quality control. Because PCCT may, in principle, provide higher quantitative accuracy due to more robust energy discrimination,^24^ we highlight results from the PCCT scanner. On this scanner, the AED and QRM phantoms both performed well, with maximum errors of 0.1 mg/mL for concentrations up to 2 mg/mL and about 0.3 mg/mL at higher concentrations. The energy dependence also closely matched that expected for iodine dissolved in water (within 7% in terms of the linear attenuation coefficient.)

To place this accuracy assessment into context, we consider the acceptance limits recommended by TG299 for quality control of synthetic iodine maps. The AED and QRM iodine inserts show close agreement between measured and specified concentrations, with errors well below the TG299 tolerance (≤20% or ≤0.5 mg/mL, whichever is greater).^2^ This makes the AED and QRM phantoms well suited for routine MECT quality control.

By comparison, the iodine‐in‐water inserts supplied with the CIRS phantom consistently underestimated the iodine concentration. On the PCCT scanner, the maximum error is about 0.3 mg/mL for specified iodine concentrations up to 2 mg/mL, increasing to as much as 3.5 mg/mL for inserts with higher specified concentrations. This makes the batch of CIRS iodine‐in‐water inserts investigated in this work inappropriate for spectral CT evaluation. Although identifying the root cause of this issue is beyond the scope of the present study, it is noteworthy that additional measurements performed with CIRS iodine‐in‐blood inserts showed an accuracy comparable to that of the other two phantoms. This suggests that the problem may be limited to the specific batch of iodine‐in‐water inserts employed.

In light of these findings, on‐site quality control of iodine inserts is recommended. The QC tests should include: (i) verifying linearity as a function of iodine concentration and (ii) verifying that the energy dependence matches that expected for iodine in water. As a simple initial check, the inserts should yield approximately 22 HU per mg/mL of iodine in a conventional polychromatic 120 kV CT acquisition (assuming water is correctly represented as 0 HU).^2^ Synthetic iodine maps and virtual monochromatic images can be used to further examine insert performance, following the methods of this work. However, the achievable accuracy is ultimately limited by the quantitative performance of the CT system, including the material decomposition algorithm used (e.g., whether a two‐ or three‐material decomposition strategy is applied). This limitation also applies to the present study, in which the inserts were evaluated using high‐dose CT acquisitions chosen to assess the phantoms' suitability for CT imaging performance studies. For applications requiring a more definitive confirmation of low iodine insert concentrations, complementary techniques such as non‐invasive x‐ray fluorescence (XRF) spectrometry may be considered.^25^

Nevertheless, for the iodine concentrations and quantitative evaluations considered in this study, the reported CT‐based measurements should be regarded as robust and relevant for routine applications. While the overall bias differed between scanners, reflecting differences in MECT implementations, the results were similar across phantoms within each scanner (AED, QRM, and CIRS with iodine‐in‐blood inserts). Moreover, results remained consistent between the body and head phantom configurations despite the large difference in their overall dimensions, indicating limited sensitivity to phantom‐ and configuration‐dependent effects such as beam hardening.

### 4.2 Tissue substitute characterization

The CIRS‐ED set of tissue substitutes showed the greatest departures from real human tissues, in terms of the EAN at a particular RED or I‐value (see Figure 6). The presence of a small amount of barium in the densest bone substitutes is known to contribute to such discrepancies.^23^ Because of this, it has previously been recommended that a direct LUT be avoided and the stoichiometric model carefully selected if using the CIRS‐ED (i.e., standard 062M) inserts.^20^, ^23^ Further, in the consensus guide of the European proton therapy community, it was recommended against using the CIRS‐ED inserts for proton therapy CT calibration at all (see the supplement to ref. [3]). However, it should be noted that all insert sets fail to deliver close tissue equivalency in terms of mass density. Because of this, although implementing a hybrid calibration for SPR and RED, the 2025 version of the consensus guide software implements a pure stoichiometric calibration for MD calibrations.

Given the lower tissue equivalency for the CIRS‐ED substitutes and recommendations against their use, it may be surprising that they provided comparable results to the other sets in Figure 7, for both SPR and RED. However, this is due to the hybrid approach of the consensus software for these two metrics.^20^ The predictions for the CIRS‐ED set are sensitive to the choice of stoichiometric model (illustrating the limitations of such models), as can be observed in Figure 8. The model of Ref. [22] has been shown to be particularly sensitive to the higher atomic number element (Ba) present in the CIRS‐ED bone substitutes.^23^ However, the hybrid consensus approach combines that stoichiometric model and a direct calibration. The overestimate from the stoichiometric model (left plot) is counteracted by an underestimation by direct calibration (right plot). This approximate cancellation also occurs for RED, but not for MD, for which a pure stoichiometric calibration is applied. This explains the trends seen in Figure 7.

Based on the results presented for tissue characterization, the AED, CIRS‐EPTN and QRM tissue substitutes can be considered to provide tissue‐equivalence for relative electron density (RED), effective atomic number (EAN) and I‐value (and hence also stopping power‐ratio). The CIRS‐ED set must be considered less satisfactory due to the composition of the bone inserts. However, all four sets show deficiencies in mass density equivalency. This may be a limitation that is difficult to overcome with polymer‐based substitute materials, although it appears feasible with liquid substitutes.^26^

From a radiation therapy perspective, the Sun Nuclear Advanced Electron Density (AED) phantom is an excellent option for CT calibration that has long been available on the market. Its satisfactory characteristics have also been demonstrated in several other studies.^3^, ^18^ The evidence of the current work indicates that the new QRM phantom is also a good choice for calibration phantom. Both the phantoms can be recommended for calibration to SPR or RED (direct, stoichiometric or hybrid methods) or to MD (stoichiometric only) and they are likely to provide results highly consistent with each other. For those centers already owning a CIRS 062M Electron Density phantom, it would be advantageous to obtain the EPTN inserts if feasible, due to the limitations with the standard CIRS electron density inserts. We note that these CIRS‐ED inserts have been used at many centers for CT calibration with the stoichiometric method.^27^ While there is no evidence that this has compromised treatment accuracy, it is clear that the other three insert sets are superior and the only ones tested here that satisfy the requirements specified in the supplement to the consensus guide to LUT generation.^3^

A limitation of the study is the reliance on vendor specifications of composition. Further limitations include the use of the ICRU tissues as reference.^8^ Typically, tissue equivalent substitutes are designed to mimic such reference tissues. These tissues represent estimates of average compositions in adults and as such do not encapsulate variations in different population groups (e.g. pediatric patients) or variations between individuals.^28^, ^29^ The choice of reference tissue compositions and CT calibration methodologies investigated here best represent current clinical practice. However, dual‐energy and photon‐counting CT techniques will in the future, when taken full advantage of, lead to a move away from the look‐up‐table approach and provide better patient specific estimations of parameters relevant to treatment planning.^13^ Representative tissue substitute sets, such as those available with Sun Nuclear's AED phantom or QRM's multi‐energy QA phantom, will nonetheless remain important, whether for calibration or validation.

## 5 CONCLUSION

The Multi‐Energy CT/Advanced Electron Density Phantom (Sun Nuclear) and the new Multi‐Energy QA phantom (QRM) are both suitable for spectral CT evaluation in diagnostic radiology as well as for CT calibration for radiation therapy treatment planning. The CIRS iodine in water inserts used in this study, however, were deemed inappropriate for spectral CT evaluation due to lower than expected iodine concentrations. Further, the tissue substitute set provided with the CIRS 062M electron density phantom was verified to be less tissue‐equivalent than the other evaluated sets, corroborating their deprecation for radiation therapy CT calibration.

## AUTHOR CONTRIBUTIONS

**Artur Omar**: Conceptualization; methodology; investigation; formal analysis; writing—original draft, writing—review & editing. **Robert Vorbau**: Methodology; investigation; formal analysis; writing—review & editing. **Jens Zimmerman**: Methodology; writing—review & editing. **Gavin Poludniowski**: Conceptualization; methodology; formal analysis; writing—original draft, writing—review & editing.

## CONFLICT OF INTEREST STATEMENT

The authors declare no conflicts of interest.

## Supporting information

Supporting Information

## Data Availability

The data that support the findings of this study are available from the corresponding author upon reasonable request.
