# Supplementary material for: Comparison of three commercial multi‐energy CT and electron density phantoms for iodine quantification and tissue substitute characterization
Source: J Appl Clin Med Phys. 2026 Aug 6;27(8):e70735. doi: 10.1002/acm2.70735 (PMC13446149; doi:10.1002/acm2.70735)
Supplement: Supplementary file 1 [file ACM2-27-e70735-s001.pdf]

Supplementary information

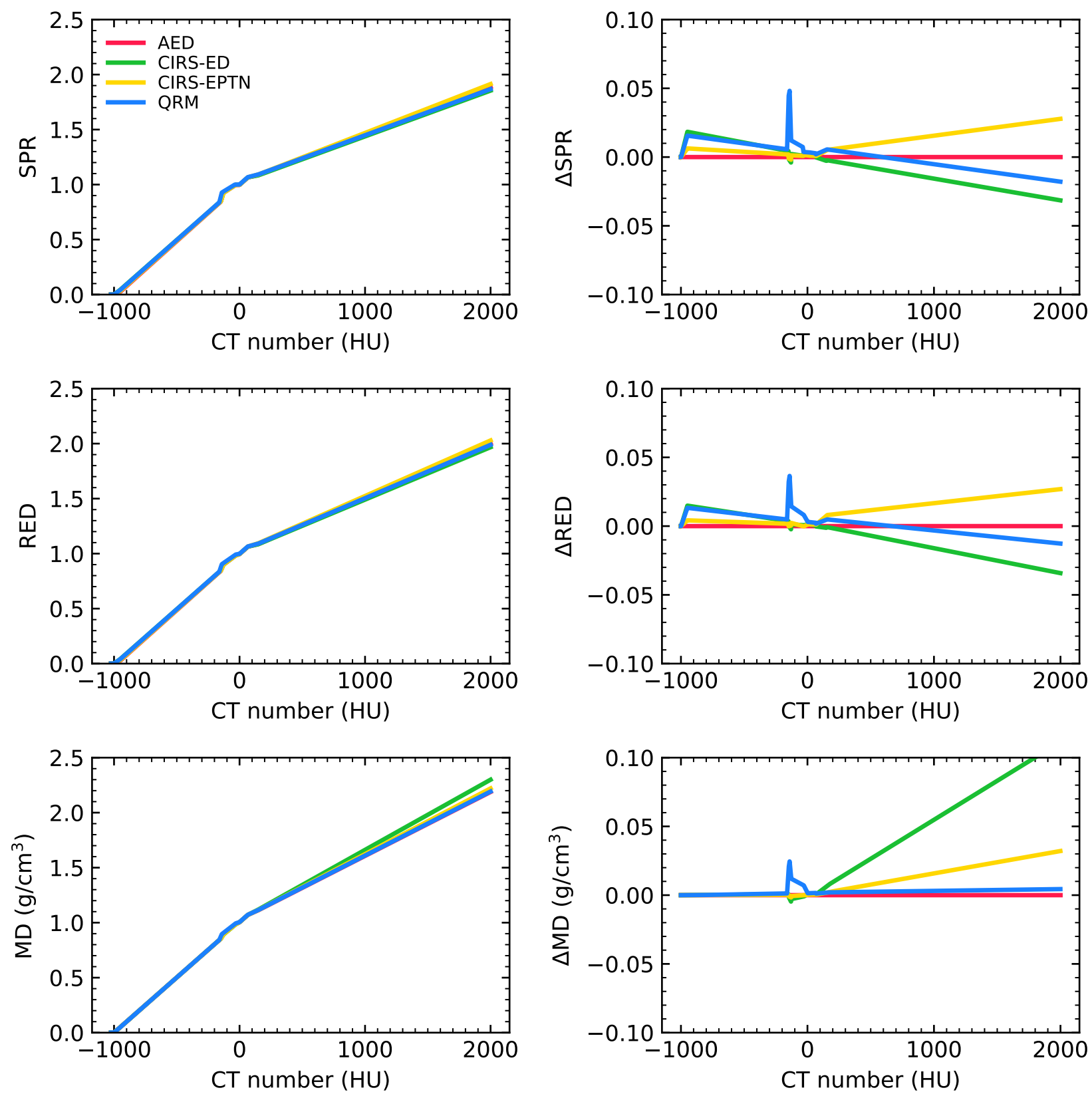

Figure S1: CT calibration look-up-tables (LUTs) for proton stopping power ratio (SPR), relative electron density (RED) and mass density (MD) generated using the consensus guide software. Results are for the SOMATOM X.ceed scanner (Siemens Healthineers, Forchheim, Germany) with 120 kV tube voltage and the four tissue substitute sets. The calibrations are for the average CT numbers of each substitute over the two phantom sizes. The left columns display the LUTs and the right column the absolute difference to a reference (AED phantom results).

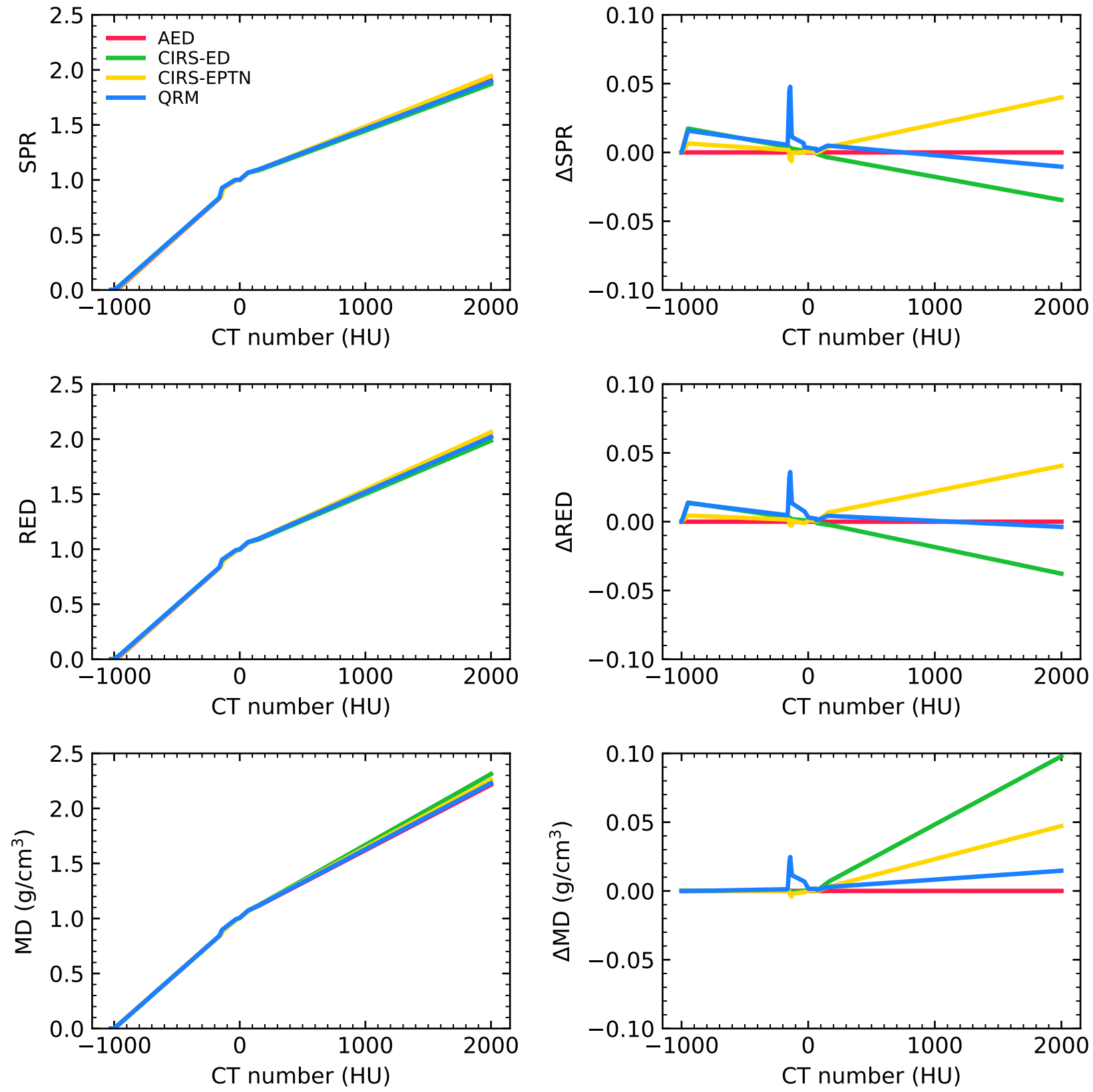

Figure S2: CT calibration look-up-tables (LUTs) for proton stopping power ratio (SPR), relative electron density (RED) and mass density (MD) generated using the consensus guide software. Results are for the Revolution Apex scanner (GE Healthcare, Waukesha, WI) with 120 kV tube voltage and the four tissue substitute sets. The calibrations are for the average CT numbers of each substitute over the two phantom sizes. The left columns display the LUTs and the right column the absolute difference to a reference (AED phantom results).
